# GB virus type C E2 protein inhibits human immunodeficiency virus type 1 Gag assembly by downregulating human ADP-ribosylation factor 1

**DOI:** 10.18632/oncotarget.6537

**Published:** 2015-12-09

**Authors:** Chenliang Wang, Christine L. Timmons, Qiujia Shao, Ballington L. Kinlock, Tiffany M. Turner, Aikichi Iwamoto, Hui Zhang, Huanliang Liu, Bindong Liu

**Affiliations:** ^1^ Center for AIDS Health Disparities Research, Department of Microbiology and Immunology, Meharry Medical College, Nashville, Tennessee, USA; ^2^ Guangdong Provincial Key Laboratory of Colorectal and Pelvic Floor Diseases, Department of Clinical Laboratory, Guangdong Institute of Gastroenterology and the Sixth Affiliated Hospital, Institute of Human Virology and Key Laboratory of Tropical Disease Control of Ministry of Education, Sun Yat-sen University, Guangzhou, Guangdong, China; ^3^ Division of Infectious Diseases, Advanced Clinical Research Center, Institute of Medical Science, University of Tokyo, Tokyo, Japan

**Keywords:** GBV-C E2, HIV-1 assembly, HIV-1 Gag, plasma membrane targeting, ARF1, Immunology and Microbiology Section, Immune response, Immunity

## Abstract

GB virus type C (GBV-C) glycoprotein E2 protein disrupts HIV-1 assembly and release by inhibiting Gag plasma membrane targeting, however the mechanism by which the GBV-C E2 inhibits Gag trafficking remains unclear. In the present study, we identified ADP-ribosylation factor 1 (ARF1) contributed to the inhibitory effect of GBV-C E2 on HIV-1 Gag membrane targeting. Expression of GBV-C E2 decreased ARF1 expression in a proteasomal degradation-dependent manner. The restoration of ARF1 expression rescued the HIV-1 Gag processing and membrane targeting defect imposed by GBV-C E2. In addition, GBV-C E2 expression also altered Golgi morphology and suppressed protein traffic through the secretory pathway, which are all consistent with a phenotype of disrupting the function of ARF1 protein. Thus, our results indicate that GBV-C E2 inhibits HIV-1 assembly and release by decreasing ARF1, and may provide insights regarding GBV-C E2's potential for a new therapeutic approach for treating HIV-1.

## INTRODUCTION

GB virus type C (GBV-C) is a single-stranded positive-sense RNA virus that belongs to the *Pegivirus* genus [1] in the *Flaviviridae* family [2]. Like HIV-1, GBV-C can be transmitted through sexual contact, blood-borne exposure, and vertically from mother to child [3]. For this reason, the prevalence of GBV-C infection is as high as 50% among high-risk populations, including HIV-1 infected patients [4]. Moreover, *in vitro* studies have shown that GBV-C replicates in lymphocytes, including CD4+ T cells, which are well-known targets for HIV-1 infection [5].

Although no evidence that GBV-C causes or promotes any human disease has been found [6], clinical and *in vitro* studies support the concept that GBV-C is associated with a delay in the progression of AIDS [reviewed in [7]]. In most studies, the beneficial effect of GBV-C viremia was found to be linked to a lower HIV-1 viral load, a higher CD4+ T cell count, reduced mortality and an improved response to highly active antiretroviral therapy (HAART) [8]. The slower HIV disease progression is primarily caused by reducing expression of the HIV entry co-receptors (CCR5 and CXCR4) and increasing secretion of chemokine ligands (MIP-1a, MIP-1b, RANTES and SDF-1) for those co-receptors.

The GBV-C E2 envelope glycoprotein, NS3 protease and phosphoprotein NS5A have been associated with the inhibitory effect of GBV-C on HIV-1 replication [9-13]. Among those GBV-C proteins, E2 was proposed to block HIV-1 entry into target cells by inhibiting gp41-mediated liposome fusion or reacting with a cellular antigen on HIV-1 particles and neutralize diverse HIV-1 isolates [10, 14, 15]. Furthermore, Bhattarai et al. showed that E2 also could disrupt T cell activation by impairing T cell receptor signaling [16]. Recently, our group showed that E2 was able to inhibit the targeting of HIV-1 Gag to the plasma membrane, which ultimately resulted in a defect in Gag assembly, precursor processing and virus release [17].

Host cellular factors are critical for retroviral Gag assembly and release [18-21]. The cellular machinery involved in the transfer of Gag through the cytosol and to the plasma membrane is not fully understood. However, clathrin-associated heterotetrameric adaptor protein (AP) complexes, suppressor of cytokine signaling 1 (SOCS1), the phospholipid, phosphatidylinositol-(4,5)-bisphosphate [PI(4, 5)P2] and ADP-ribosylation factor (ARF) are implicated in this process [reviewed in [22]].

ARF proteins regulate a variety of membrane trafficking pathways. They are divided into three classes. Class I ARFs (ARF 1-3) regulate the assembly of coat protein complexes in the secretory pathway. Class II ARFs (ARF 4-5) function in protein and vesicle transport in the Golgi, while Class III ARFs (ARF 6) serve roles in actin remodeling and endocytic membrane trafficking [23-25]. Interestingly, Joshi et al. reported that knocking down ARF1 interfered with Gag membrane association and led to the accumulation of intracellular Gag, which caused an inhibitory effect of HIV-1 virus release. The functions of ARF1 and other ARF proteins were found to be critical for Gag plasma membrane localization and Gag particle production [26].

In the present study, we identified ARF1 as a cellular factor contributing to the inhibitory effect of GBV-C E2 on HIV-1 Gag membrane targeting. Our results indicate that GBV-C E2 inhibited HIV-1 Gag targeting to the plasma membrane by decreasing protein level of ARF1 through the proteasomal degradation pathway. Restoration of ARF1 expression rescued the HIV-1 Gag processing and membrane targeting defect imposed by GBV-C E2 expression. The decreased ARF1 expression by GBV-C E2 was also confirmed by confocal microscopy studies showing a disruption in Golgi morphology and trafficking to and from the Golgi-derived vesicles. This work reveals the mechanism by which GBV-C E2 inhibits HIV-1 assembly and release, as well as the interaction between GBV-C E2 and the human ARF protein system.

## RESULTS

### Expression of GBV-C E2 downregulates ARF1 protein expression without inhibiting ARF1 transcription

**Figure 1:**
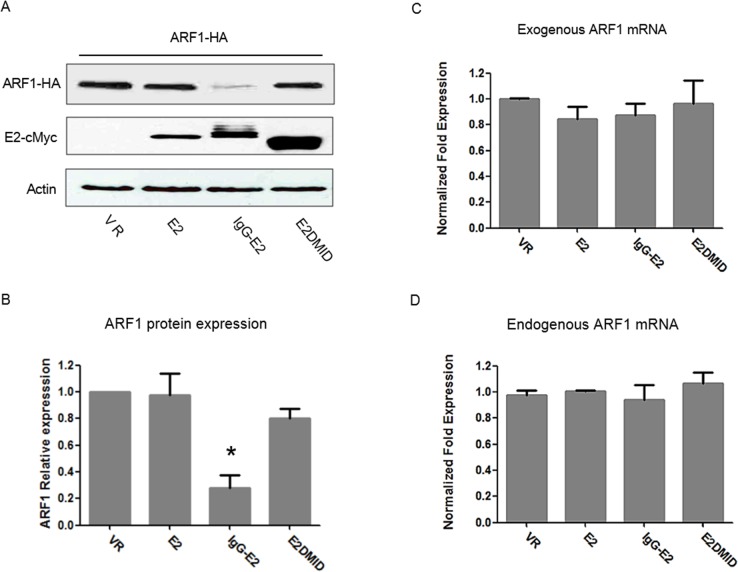
Expression of GBV-C E2 downregulates ARF1 protein expression without inhibiting ARF1 transcription **A.** VR1012, E2, IgG-E2 or E2DMID expression vector was co-transfected with the ARF1-HA expression vector into 293T cells. Cell lysates were subjected to Western blotting analysis. **B.** Relative ARF1 protein expression was determined by densitometry analysis. The data are represented as mean ± SD, *P < 0.05, one-way ANOVA. Total RNA isolated from the transfected samples were subjected to real-time PCR to determine **C.** exogenous *ARF1* mRNA levels and **D.** endogenous *ARF1* mRNA levels. The data are represented as mean ± SD. All the values are from the average of three independent experiments.

E2 is predicted to be expressed in a glycosylated form and targeted to the endoplasmic reticulum during GBV-C replication. Thus, the secretory signal peptide of immunoglobulin G (IgG) was fused to the N-terminus of E2 to create a glycosylated E2 expression construct (IgG-E2). We previously showed that the GBV-C envelope glycoprotein E2 (IgG-E2) could inhibit HIV-1 assembly and release by disrupting the targeting of HIV-1 Gag to the plasma membrane [17]. As the ARF proteins were reported to facilitate Gag-membrane binding [26], we decided to examine whether the E2 expression has an effect on ARF proteins and mRNA levels. Since ARF1 is the most abundant, active and best-characterized ARF family protein [reviewed in [24]], we will focus on studying the interplay between IgG-E2 and ARF1 in this study. The unglycosylated E2 and E2DMID, which had no effect on HIV-1 assembly and release in our prior study [17], were used as a control in this study. Both IgG-E2 and E2DMID contain an IgG signal peptide at the N-terminus, and the only difference between them is the internal deletion of the membrane interaction domain (MID) region of IgG-E2 in the E2DMID construct. Western blotting analysis revealed that the co-expression of IgG-E2 led to a five-fold decrease in ARF1 expression, compared with the VR1012, E2 or E2DMID (Figure 1A, 1B, *P* = 0.022). The mRNA levels of exogenous and endogenous *ARF1* did not decrease with IgG-E2 expression (Figure 1C, 1D). These data suggest that IgG-E2 expression downregulates ARF1 protein expression without inhibiting ARF1 transcription.

### Expression of GBV-C E2 reduces ARF1 protein half-life

**Figure 2:**
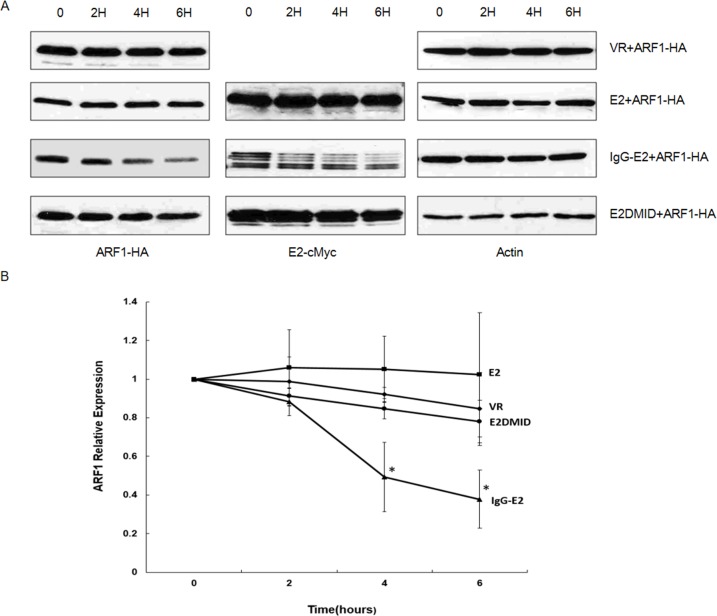
GBV-C E2 reduces ARF1 stability **A.** VR1012, E2, IgG-E2 or E2DMID expression vector was co-transfected with the ARF1-HA expression vector into 293T cells. Twenty-four hours post-transfection, cells were treated with CHX (200 μg/ml) and then harvested at the indicated time points for subsequent analysis by Western blotting. **B.** Relative ARF1 protein expression was quantified by densitometry analysis. The data are represented as mean ± SD, *P < 0.05, Student's *t*-test. All the values are from the average of three independent experiments.

We then studied the effects of IgG-E2 on the half-life of ARF1 protein. The ARF1 expression vector was co-transfected with VR1012, E2, IgG-E2 or E2DMID into 293T cells. Twenty-four hours post-transfection, the cells were treated with the protein synthesis inhibitor cycloheximide (CHX) and harvested at the indicated time points to measure ARF1 expression by Western blotting analysis. As shown in Figure 2, the levels of ARF1 protein decreased by about 50% in 4 h in the presence of IgG-E2 (*P* = 0.017). By contrast, the protein level of ARF1 remained almost unchanged for up to 6 h when ARF1 was co-expressed with the VR1012, E2 or E2DMID. From this analysis, we concluded that IgG-E2 reduces the half-life of the ARF1 protein.

### Maintaining steady state level of ARF1 expression compromises the inhibitory effect of GBV-C E2 on HIV-1 Gag processing

**Figure 3:**
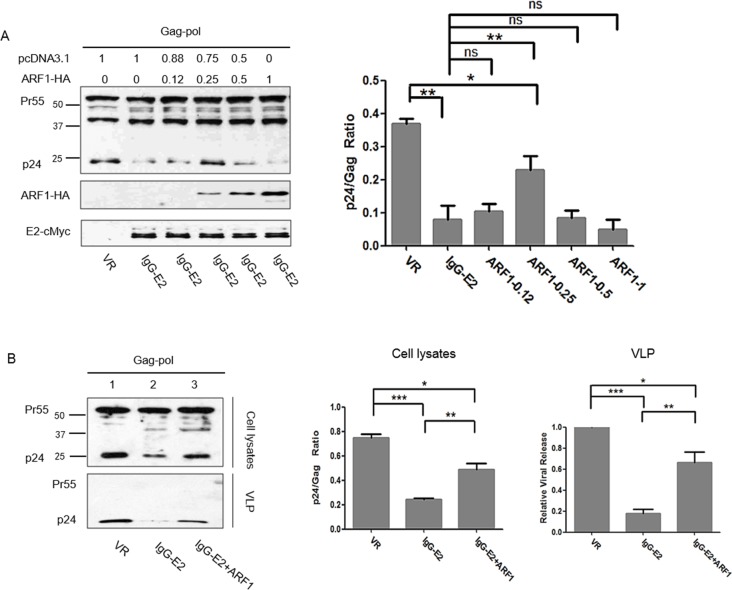

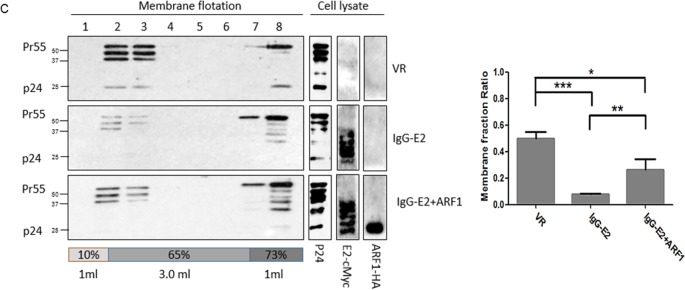
Maintaining ARF-1 expression at a steady state level compromises the inhibitory effect of GBV-C E2 on HIV-1 Gag processing **A.** ARF1-HA was co-transfected with Gag-Pol and IgG-E2 into 293T cells at indicated amounts, cells were harvested for Western blotting analysis, and the ratio of p24 to Gag (all Gag-related proteins except p24) in cell lysates was determined by densitometry analysis. The data are represented as mean ± SD, *P < 0.05, **P < 0.01, ns indicates no significant difference, Student's *t*-test. **B.** The Gag-Pol expression vector was co-transfected with VR1012, IgG-E2 or IgG-E2 with 0.25 μg of the ARF1-HA expression construct, cells were either lysed for Western blotting analysis or sonicated for the membrane flotation assay. The culture supernatant was subjected to ultracentrifugation for measuring VLP release. Relative VLP release, determined by densitometry analysis, was calculated as the released Gag divided by the total Gag from cell lysate and virions. The data are represented as mean ± SD, *P < 0.05, **P < 0.01, ***P < 0.001, Student's *t*-test. **C.** Sonicated cell lysates were subjected to membrane flotation analysis. Eight fractions were collected from each sample after ultracentrifugation. The fraction samples and total cell lysates were analyzed by Western blotting. The data are represented as mean ± SD, *P < 0.05, **P < 0.01, ***P < 0.001, Student's *t*-test. All the values are from the average of three independent experiments.

We next examined the effect of GBV-C E2 on the plasma membrane targeting and processing of Gag in the presence of increasing amounts of ARF1. Different amounts of ARF1 expression vector were therefore co-transfected with the Gag-Pol expression vector and IgG-E2 plasmid as indicated in Figure 3A. Forty-eight hours post-transfection, cell lysates were subjected to Western blotting analysis. Transfecting with 0.25 μg of ARF1-HA abrogated the inhibition of Gag processing by IgG-E2 (Figure 3A, *P* = 0.0063), while using 0.5 μg or 1 μg of ARF1-HA restored the sensitivity of HIV-1 Gag to IgG-E2 (Figure 3A). This result suggests that a certain steady state level of ARF1 was required in order to perform its normal function. To investigate whether maintaining the steady state level of ARF1 could inhibit the effect of IgG-E2 on Gag VLP release, the Gag-Pol expression construct was co-transfected with VR1012, IgG-E2 or IgG-E2 with 0.25 μg of the ARF1 expression construct. Forty-eight hours after transfection, the culture supernatants were harvested for collection of VLPs by ultracentrifugation as described in Materials and Methods. The relative viral release was calculated as the released Gag divided by the total Gag from the cell lysate and virions. As shown in Figure 3B, ARF1 expression significantly compromised the IgG-E2 inhibitory effect on Gag-Pol VLP release (Figure 3B, lane 3 *vs*. lane 2, *P* = 0.0015). We also performed this experiment using Jurkat cells, a more physiologically relevant CD4+ T cells line. As seen in Figure S1, similar results were obtained in Jurkat cells.

In our previous work, we showed that the expression of IgG-E2 could inhibit Gag membrane targeting using a membrane flotation assay [17]. Thus, here we tested whether ARF1 expression could rescue the Gag membrane targeting defect caused by IgG-E2 using the same method. In this assay, membrane-associated proteins migrated to the interface between 65% and 10% sucrose cushions (Figure 3C). When Gag-Pol was expressed alone with VR1012, significant amounts of Gag (∼56%) were localized in fraction 2 and 3, which represent the membrane-associated Gag. By contrast, in cells expressing IgG-E2, only a small amount of the Gag protein (∼8%) was localized in membrane-associated fractions. Meanwhile, in cells co-expressing IgG-E2 with the ARF1 expression construct, membrane-associated Gag was increased by about two-fold (∼26%) compared with those expressing IgG-E2 alone (*P* = 0.0070). These results suggest that the expression of ARF1 partially restored Gag membrane targeting in the presence of IgG-E2 expression.

**Figure 4:**
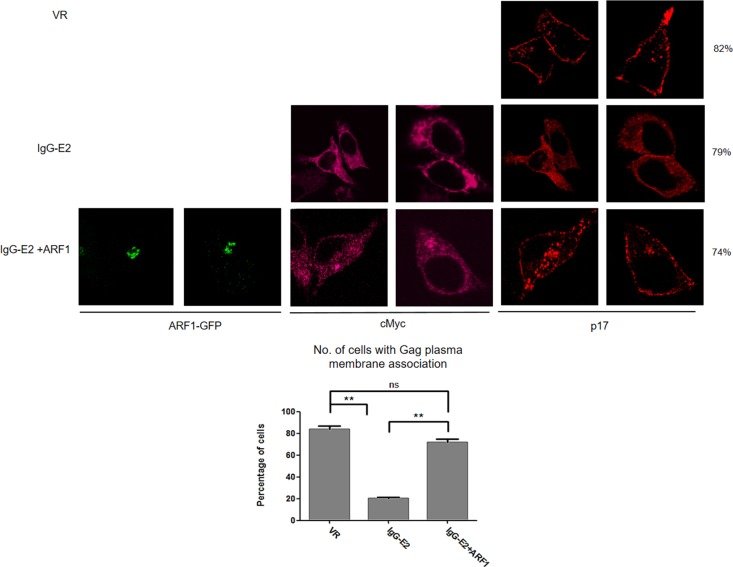
Maintaining ARF-1 expression at a steady state level compromises the inhibitory effect of GBV-C E2 on HIV-1 Gag targeting to the plasma membrane The Gag-Pol expression construct was co-transfected with VR1012, IgG-E2 or IgG-E2 with an ARF1-GFP expression construct into HeLa cells. Cells were fixed, permeabilized and immunostained with anti-HIV-1 p17 polyclonal (Red) antisera, anti-cMyc antibody (Peachpuff). Fifty cells were counted for each sample. The percentage of cells, which showed the presented pattern were calculated and were shown at the far right side of the figure. The data are represented as mean ± SD, **P < 0.01, Student's *t*-test. All the values are from the average of three independent experiments.

We next sought to evaluate whether ARF1 expression rescues the Gag membrane targeting defect by using immunofluorescence microscopy. The Gag-Pol expression construct was transfected into HeLa cells, along with VR1012, IgG-E2 or IgG-E2 plus ARF1 expression vectors ARF1-GFP. The cells were stained with antibodies directed against Gag-p17 for analysis of Gag localization. IgG-E2 was stained with a monoclonal anti-cMyc antibody. In the absence of IgG-E2, Gag distribution was punctated and localized primarily toward the periphery along the plasma membrane (Figure 4, top row), in agreement with previous reports [27-29]. By contrast, in the presence of IgG-E2, Gag was predominantly found throughout the cytoplasm (Figure 4, middle row). However, when IgG-E2 was co-expressed with ARF1, the distribution of Gag was then shown to be punctated and restored to the area around the plasma membrane (Figure 4, bottom row). These data indicate that the expression of ARF1 restored Gag trafficking to the plasma membrane, once again supporting the concept that GBV-C E2 inhibits Gag assembly and release by downregulating ARF1.

### GBV-C E2 induces ARF1 degradation through the proteasomal degradation pathway

**Figure 5:**
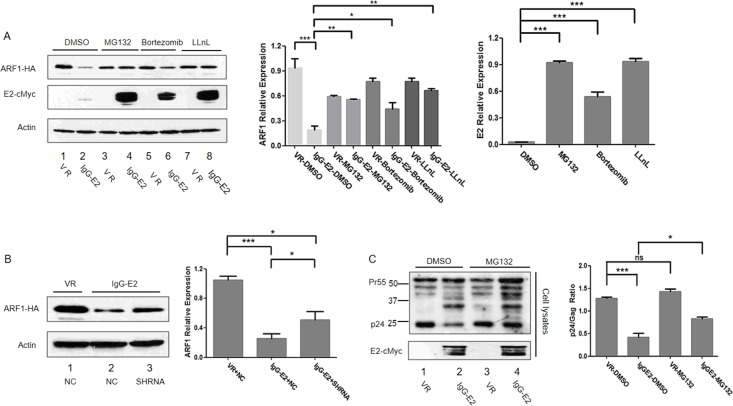
GBV-C E2 induces ARF1 degradation through the proteasomal degradation pathway **A.** 293T cells were transfected with ARF1-HA and either VR1012 or IgG-E2. Proteasomal inhibitors MG132 (10 μM), bortezomib (50 nM) or LLnL (50 μM) were added to the culture medium for 16 h. Cells were harvested for Western blotting analysis. The data are represented as mean ± SD, *P < 0.05, **P < 0.01, ***P < 0.001, Student's *t*-test. **B.** ARF1-HA was co-transfected with VR1012 plus NC, IgG-E2 plus NC or IgG-E2 plus PMSA1-targeted shRNA into 293T cells. Cell lysates were analyzed by Western blotting. Relative ARF1 protein expression was determined by densitometry analysis. The data are represented as mean ± SD, *P < 0.05, ***P < 0.001, Student's *t*-test. **C.** 293T cells were co-transfected with Gag-Pol and VR1012, IgG-E2. The proteasomal inhibitor MG132 (10 μM) was added to treat the cells for 16 h. Cell lysates were harvested for Western blotting. Ratios of p24 to Gag (all Gag related proteins except p24) in cell lysates were determined by densitometry analysis. The data are represented as mean ± SD, *P < 0.05, ***P < 0.001, Student's *t*-test. All the values are from the average of three independent experiments.

Thus far, we found that IgG-E2 abolished ARF1 expression (Figures 1 and 2), which was required for IgG-E2 to inhibit HIV Gag processing (Figures 3 and 4). Since the proteasomal degradation pathway is a major cellular pathway for protein turnover, we decided to test whether IgG-E2 decrease ARF1 expression through the proteasomal degradation pathway. ARF1-HA was co-transfected with either VR1012 or IgG-E2 expression constructs into 293T cells. Twenty-four hours post-transfection, the cells were treated with either DMSO or the 26S proteasome inhibitor MG132 (also referred to as aldehyde N-carbobenzoxy-L-leucyl-L-leucyl-L-leucinal or nLLL), bortezomib or calpain inhibitor 1 (LLnL) for 16 h before Western blotting analysis. As expected, when cells were treated with DMSO, IgG-E2 reduced ARF1-HA expression, compared to control samples (Figure 5A, lane 2 *vs*. lane 1, *P* < 0.001). By contrast, MG132, bortezomib and LLnL treatment prevented degradation of ARF1 by IgG-E2 (Figure 5A, lanes 4, 6 and 8 *vs*. lane 2, *P* = 0.0030, 0.018, 0.0017 respectively) even though bortezomib was less potent than MG132 and LLnL (Figure 5A lane 6 *vs*. lanes 4 and 8). These results imply that IgG-E2 induces ARF1 degradation through the proteasomal pathway. IgG-E2 expression was noted to be also significantly increased with MG132, bortezomib and LLnL treatment (Figure 5A, lanes 4, 6 and 8 *vs*. lane 2, *P* < 0.001), suggesting that IgG E2 was co-degraded with ARF1 by the proteasomal degradation pathway. Once the proteasomal degradation pathway was blocked by the inhibitors, both ARF1 and IgG-E2 increased.

PMSA1 is an essential functional component of the proteasomal complex, and its knockdown was shown to inhibit the proteasomal degradation pathway [30, 31]. As shown in Figure 5B, when the PMSA1-targeted shRNA was introduced into the IgG-E2 and ARF1 co-transfected cells, the PMSA1-targeted shRNA also partially blocked IgG-E2-induced ARF1 degradation (Figure 5B, lane 3 *vs*. lane 2, *P* = 0.034). This result further suggests a role for the proteasome in the IgG-E2-induced degradation of ARF1.

Since a proteasomal inhibitor blocked IgG-E2-induced ARF1 degradation, we were interested in determining whether MG132 could rescue the HIV-1 Gag processing defect induced by IgG-E2. For this study, Gag-Pol was co-transfected with VR1012 or IgG-E2 into 293T cells. At 24 h post-transfection, cells were treated for 16 h with MG132 or DMSO as a control before Western blotting analysis. As expected, Gag processing was aberrant in the DMSO control group in the presence of IgG-E2 (Figure 5C, lane 2 *vs*. lane 1). However, Gag processing was partially restored when MG132 was used to treat the cells (Figure 5C, lane 4 *vs*. lane 2, *P* = 0.014). These results further suggest that GBV-C E2 inhibits HIV Gag processing by downregulating ARF1 expression.

### IgG-E2 expression causes a change in golgi morphology

**Figure 6:**
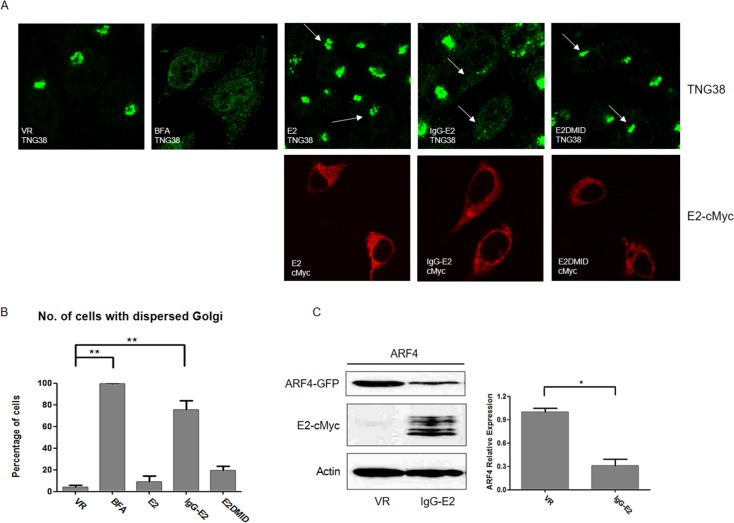
GBV-C E2 expression causes the collapse of the Golgi complex **A.** HeLa cells were transfected with VR1012, E2, IgG-E2 or E2DMID. One VR1012 sample group was treated with BFA (5 μg/ml) for 30 min, and then all cells were fixed, permeabilized and immunostained. Golgi and E2 proteins were stained with anti-TGN38 (Green) and anti-cMyc (Red) antibodies respectively. Arrows are used to indicate those cells expressing E2, IgG-E2 or E2DMID. **B.** Fifty cells were counted in each sample, and the percentage of cells with a dispersed Golgi pattern was calculated and is shown in the bar chart. The data are represented as mean ± SD, **P < 0.01, Student's *t*-test. **C.** VR1012 or IgG-E2 expression vector was co-transfected with ARF4-GFP expression vector into 293T cells. Cells were harvested for Western blotting analysis. The data are represented as mean ± SD, *P < 0.05, Student's *t*-test. All the values are from the average of three independent experiments.

ARF proteins play a pivotal role in maintaining Golgi morphology [32, 33]. ARF inhibitor Brefeldin A (BFA) treatment and downregulation of ARF1 together with ARF4 expression have been shown to cause dramatic changes in Golgi morphology [34]. As we showed that IgG-E2 abrogated ARF1 expression, we were interested in determining whether the expression of IgG-E2 could alter the morphology of the Golgi. Accordingly, we transfected HeLa cells with VR1012, E2, IgG-E2 or E2DMID. BFA-treated HeLa cells (VR1012 transfected) were used as a positive control. As exhibited by immunofluorescent staining of the Golgi resident protein TNG38, expression of IgG-E2 caused the Golgi to disperse throughout the cell in a manner which was indistinguishable from the BFA-treated cells (Figure 6A, top fourth from the left *vs*. top second from the left). About 80% of cells expressing IgG-E2 showed Golgi morphological changes, whereas ∼20% of cells expressing E2DMID and < 10% of cells expressing E2 displayed any changes in Golgi morphology, compared to cells transfected with the VR1012 control (Figure 6B, VR *vs*. BFA, *P* = 0.0019, VR *vs*. IgG-E2, *P* = 0.0022). Both IgG-E2 and E2DMID contain an IgG signal peptide at the N-terminus. Therefore, these results further confirm that the effects of GBV-C E2 in disrupting Golgi morphology were determined by the MID domain but not the IgG signal peptide. Here, we demonstrated that IgG-E2 expression altered Golgi morphology in a manner that was similar to that observed when BFA was used to treat the cells, or when ARF1 and ARF4 functions were impaired [34]. In those studies, only double knockdown of both ARF1 and ARF4 caused a Golgi morphological change, while the single knockdown of ARF1 or ARF4 was ineffective [34], suggesting that IgG-E2 expression may downregulate ARF4 expression as well. Indeed, we independently observed that IgG-E2 downregulated ARF4 expression (Figure 6C, right panel, *P* = 0.020).

### IgG-E2 expression alters protein trafficking through the secretory pathway

**Figure 7:**
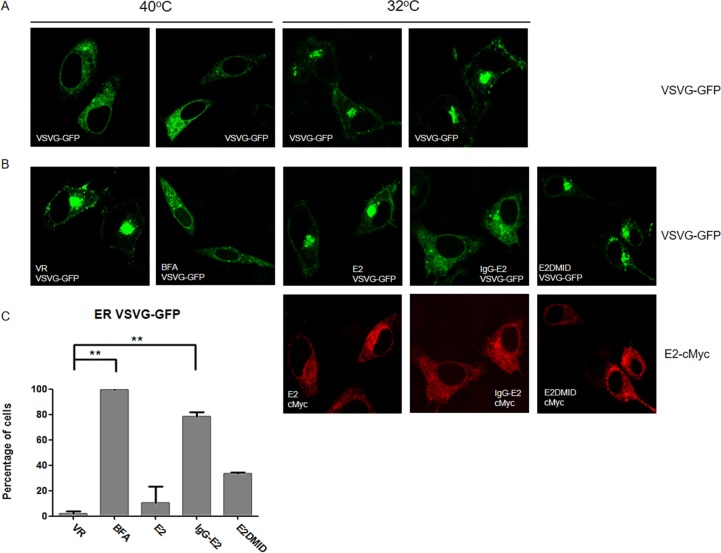

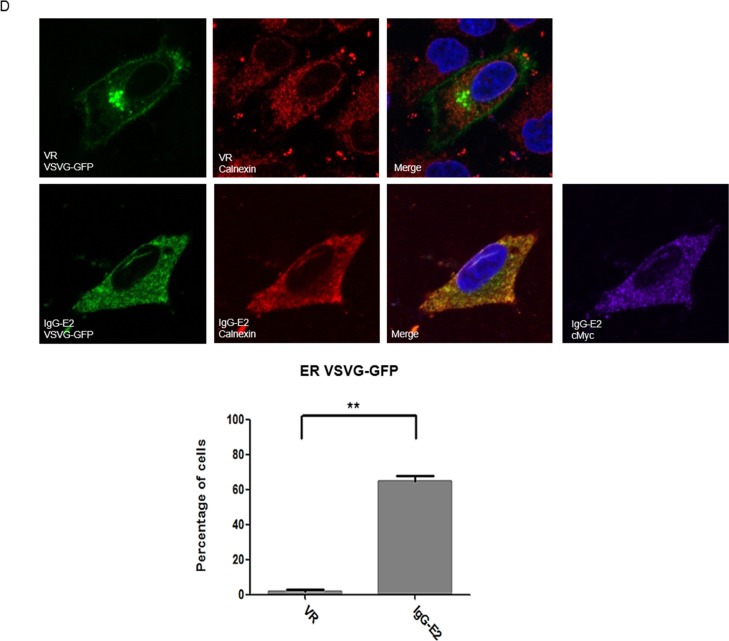

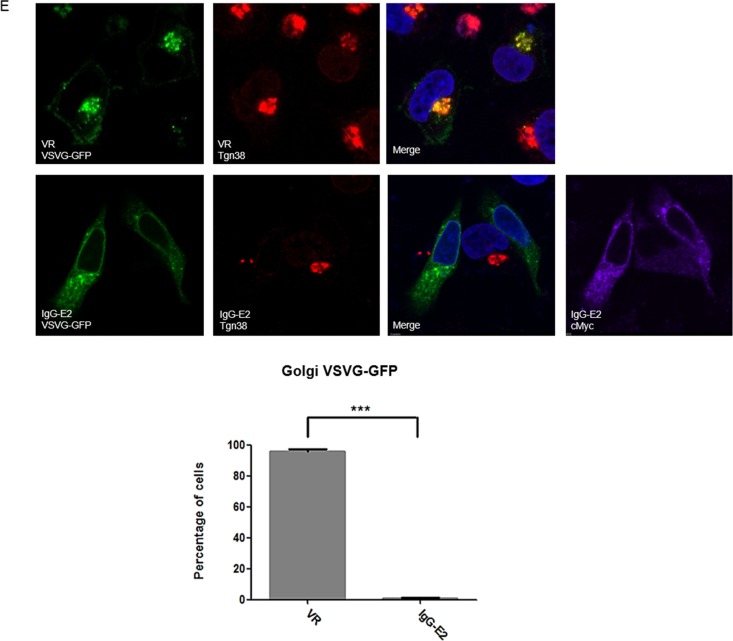
GBV-C E2 expression alters trafficking through the secretory pathway **A.** HeLa cells were transfected with the ts045-VSVG-GFP expression vector and incubated at 37°C for 6 h, and then the medium was replaced with fresh medium. After incubation at 40°C for 16 h, cells were either fixed immediately or switched to 32°C for 90 min and then fixed. **B.** The expression vector ts045-VSVG-GFP was co-transfected with VR1012, E2, GBV-C E2 or E2DMID into HeLa cells and incubated at 37°C for 6 h, and then the medium was replaced with fresh medium. After incubation at 40°C for 16 h, one group of cells transfected with VR1012 was treated with BFA (5 μg/ml) for 30 min, and then all cells were switched to 32°C for 90 min before they were fixed, permeabilized and immunostained. E2 proteins were stained with the anti-cMyc antibody (Red). **C.** Fifty cells were counted in each sample, and the percentage of cells showing the perinuclear VSV-G pattern was calculated and shown in the bar chart. Statistical analysis was performed. The data are represented as mean ± SD, **P < 0.01, Student's *t*-test. **D.** and **E.** The expression vector ts045-VSVG-GFP was co-transfected with VR1012 or IgG-E2 into HeLa cells and incubated at 37°C for 6 h, and then the medium was replaced with fresh medium. After incubation at 40°C for 16 h, all cell samples were cultured in 32°C for 90 min before they were fixed, permeabilized and immunostained as described in Materials and Methods. E2 proteins were stained with the anti-cMyc antibody, ER was stained with the anti-calnexin antibody (D) and Golgi was stained with the anti-TGN38 antibody (E). DAPI was used to stain the nuclei. ER (D) and Golgi (E) are shown in red, VSV-G is shown in green and E2 protein is shown in purple. Fifty cells were counted in each sample, and the percentage of cells showing the perinuclear VSV-G pattern was calculated and shown in the bar chart. Statistical analysis was performed. The data are represented as mean ± SD, **P < 0.01, ***P < 0.001, Student's *t*-test. All the values are from the average of three independent experiments.

In addition to impairing Golgi morphology, ARF1 mutants and BFA treatment have been shown to inhibit trafficking of proteins through the early secretory pathway [35-39]. To determine the effect of IgG-E2 on early secretory trafficking, we analyzed green fluorescent protein (GFP) tagged vesicular stomatitis G protein (VSV-G) trafficking through the secretory pathway in the presence or absence of IgG-E2 expression. The form of VSV-G used in this system was a temperature sensitive mutant, ts045-VSVG-GFP [36, 40]. At 40°C, the VSV-G mutant was misfolded and retained in the endoplasmic reticulum (ER) (Figure 7A, first and second from the left). After transferring to the permissive temperature of 32°C, the VSV-G mutant was properly folded and transported to Golgi (Figure 7A, third and fourth from the left). To study the effect of IgG-E2 on the secretory pathway, we co-transfected the ts045-VSVG-GFP mutant with empty vector VR1012, E2, IgG-E2 or E2DMID into HeLa cells. HeLa cells transfected with ts045-VSVG-GFP mutant alone were treated with BFA as a positive control. The ts045-VSVG-GFP transport assay was performed as described in the Material and Methods section. As expected, the VSV-G mutant was transported to the Golgi (Figure 7B, top first from the left), while almost all of the VSV-G was trapped in the ER with BFA treatment (Figure 7B, top second from the left). The expression of E2 or E2DMID had either no or a minor effect on VSV-G mutant trafficking, and most of the cells showed that the VSV-G mutant was transported to the Golgi (Figure 7B, top third and fifth from the left). On the other hand, around 80% of cells expressing IgG-E2 showed VSV-G mutant distributed in an intracellular punctated pattern or dispersed throughout the cytoplasm (Figure 7B, top fourth from the left, *P* = 0.0016), which confirmed that the VSV-G mutant co-localized with the ER (Figure 7D 2^nd^ row, *P* = 0.011) and not the Golgi anymore (Figure 7E 2^nd^ row, *P* < 0.001). These data indicate that IgG-E2 expression disrupts trafficking through the secretory pathway.

### IgG-E2 expression does not cause significant cytotoxicity

**Figure 8:**
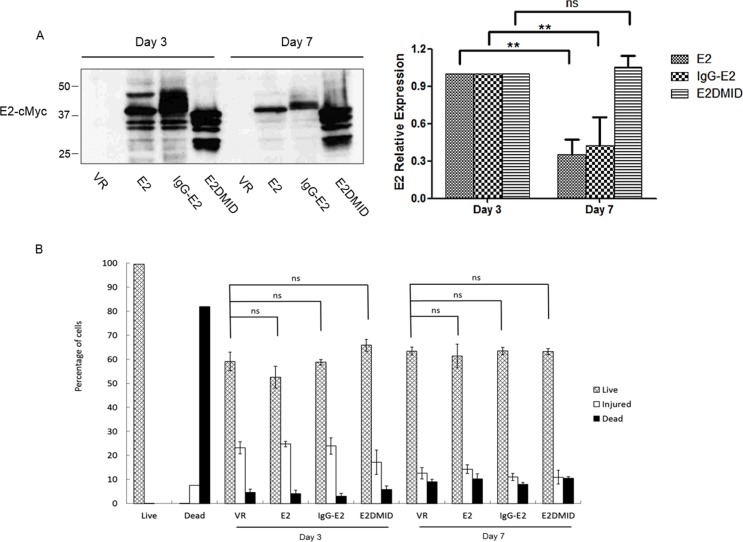
Expression of IgG-E2 does not cause dramatic cytotoxicity **A.** 293T cells were transfected with VR1012, E2, IgG-E2 or E2DMID. At 3 and 7 days post-transfection, cells were harvested for Western blotting analysis. The data are represented as mean ± SD, **P < 0.01, ns indicates no significant difference, Student's *t*-test. B. Transfected cells were also stained by using the LIVE/DEAD^®^ Cell Vitality Assay kit. Cell viability was analyzed using a BD Biosciences FACScalibur with excitation at 488 nm, and the fluorescence emission was measured at 530 nm and 575 nm. The data are represented as mean ± SD, ns indicates no significant difference, Student's *t*-test. All the values are from the average of three independent experiments.

As shown in Figures 6 and 7, IgG-E2 expression caused dramatic changes in Golgi morphology and inhibition of protein trafficking through secretory pathway. To determine whether the expression of IgG-E2 might cause cytotoxicity, VR1012, E2, IgG-E2 or E2DMID was transfected into 293T cells. On day 3 and 7, cell viability was measured by the LIVE/DEAD Cell Vitality Assay. Western blotting analysis was used to measure protein expression. As shown in Figure 8A, the transient expression of E2 and IgG-E2 reached the peak on day 3 and then significantly decreased on day 7 (Figure 8A, E2, *P* = 0.0071; IgG-E2, *P* = 0.0090). This result further suggests that E2 was degraded in the cells. By contrast, the expression of E2DMID stayed unchanged during the 7-day culture (Figure 8A, E2DMID, *P* = 0.21), implying that the MID was the determinant for the E2 degradation. During the 7-day cell culture, we did not observe a significant difference in the numbers of live, injured and dead cells among the VR1012, E2, IgG-E2 and E2DMID samples. Results of LIVE/DEAD Cell Vitality Assay showed that the exogenous protein expression did not cause significant cell injury or death to 293T cells comparing to mock-transfected cells during a 7-day tissue culture system (Figure 8B). A similar data was obtained with SupT1 cells (Figure S3).

## DISCUSSION

The GBV-C E2 envelope glycoprotein has been shown to be at least partially responsible for the GBV-C inhibitory effect on HIV-1 replication [41]. The evidence includes the fact that HIV-1 entry is blocked by extracellular exposure to GBV-C E2 or intracellularly expressed GBV-C E2 [9, 42]. We recently showed that GBV-C E2 inhibits HIV-1 assembly and release by inhibiting HIV-1 Gag targeting to the plasma membrane [17]. Because we did not detect a direct interaction between HIV-1 Gag and GBV-C E2 (data not shown), we speculated that some host cellular factors may play a role in the GBV-C E2-mediated inhibitory effect on Gag targeting to the plasma membrane. In this study, we observed that expression of GBV-C E2 decreased the expression of ARF1, which is critical for Gag assembly and release [26]. The decrease in ARF1 expression was attributed to a reduction in ARF1 protein stability, rather than a reduction in transcription or the stability of ARF1 mRNA (Figure 1C, 1D). As seen in Figure 3 and Figure 4, restoration of ARF1 expression can rescue the HIV-1 Gag processing and membrane binding defect imposed by IgG-E2 expression. Observations with the proteasomal degradation inhibitors and shRNA knockdown of the PMSA1 (Figure 5) suggest that IgG-E2 induced ARF1 degradation through a proteasomal degradation pathway. Taken together, these data suggest that GBV-C E2 inhibits HIV-1 Gag processing and release by downregulating ARF1 expression.

One interesting point is that a certain steady state level of ARF1 was required in order to support Gag processing (Figure 3A). This phenotype is consistent with that of some other cellular factors, such as Tsg101, which are involved in HIV-1 replication. When the expression of Tsg101 was downregulated, a late domain defect would occur in HIV-1 assembly [21]. However, if Tsg101 was overexpressed, it would cause a viral assembly defect as well [43], suggesting a steady state level of Tsg101 is required for supporting HIV assembly. In the case of ARF1, when we overexpressed ARF1 (Figure 3A, 0.5-1μg ARF1), ARF1 inhibited HIV Gag processing. It is consistent with previous reports that an imbalance in ARF1 level leads to the inhibition of ARF1 function [36, 44, 45].

We demonstrated that IgG-E2 expression caused dramatic alterations in Golgi morphology and trafficking through the secretory pathway (Figure 6 and Figure 7). The phenotypes mediated by IgG-E2 expression were similar to that observed in cells treated with ARF1 inhibitor BFA or in cells expressing mutant forms of ARF1 [32, 33]. These data further confirm the influence of GBV-C E2 on ARF1 degradation. Surprisingly, although GBV-C E2 expression altered Golgi morphology and the host protein secretory pathway, we did not observe a dramatic cytotoxic effect of GBV-C E2 expression in 293T cells (Figure 8) and SupT1 cells (Figure S3). In our prior study, we also did not detect significant cytotoxicity when different doses of the IgG-E2 expression vector were transfected into 293T cells [17]. Thus, these cells tolerated GBV-C E2 expression, which coincides with the notion that GBV-C is a non-pathogenic human virus [6].

Although the ARFs family proteins (except ARF2) are ubiquitously expressed in human cells, ARF1 is the most abundant, active and best-characterized ARF family protein [reviewed in [24]]. Even though we focused on ARF1 in the present study, we don't rule out the possibility that GBV-C E2 may affect other ARF isoforms. Indeed, our data showed that GBV-C E2 also downregulated ARF4 expression (Figure 6). It would be interesting to examine whether GBV-C E2 affects the expression of other ARF proteins, which might also contribute to the regulation of Gag processing.

Since GBV-C co-infection with HIV initially was associated with a delay in the progression of AIDS in clinical investigations, several mechanisms have been proposed to explain this effect, such as GBV-C infection downregulating HIV entry co-receptors CCR5 and CXCR4 and increasing the secretion of their ligands RANTES, MIP-1α, MIP-1β and SDF-1 [46]. Furthermore, *in vitro* GBV-C E2 antibodies were found to immunoprecipitate HIV particles and inhibit HIV replication [15], and GBV-C E2-derived peptides could inhibit HIV fusion [14]. GBV-C NS3 was also shown to inhibit the interferon response, which is associated with clinical benefits in AIDS patients [47]. During the preparation of this manuscript, a report was published suggesting that GBV-C decreases inflammation and improves HIV disease outcome in individuals co-infected with GBV-C and HIV [48].

The finding in this study adds another possible mechanism by which GBV-C E2 inhibits HIV-1 assembly and release through downregulation of human ARF1, a critical host factor involved in HIV assembly and release. Our work further confirms the involvement of ARF1 in HIV assembly and release, and it also implicates the role of GBV-C in the interference with HIV-1 replication and provides insights regarding the potential for a new therapeutic approach for treating HIV-1. Indeed, as with some of the models put forth earlier, the action of our proposed mechanism requires GBV-C and HIV-1 to infect the same cells, which is theoretically possible even though by chance would seem to occur at a low rate. However, the precise rates of GBV-C and HIV-1 co-infected cells in patients and especially the specific interactions and consequences of such co-infections in different compartments of the body are not well defined. Clearly, more physiologically relevant investigations are needed to determine the dominant mechanism(s) involved in the effect of GBV-C inhibiting AIDS progression.

## MATERIALS AND METHODS

### Plasmids, antibodies and reagents

GBV-C E2, IgG-E2, E2DMID, and Gag-Pol expression constructs were previously described [17, 49]. GBV-C E2 is natively expressed as a glycoprotein. Based on the amino acid sequence similarity between GBV-C and HCV, the C-terminus of GBV-C E1 has been predicted to contain the secretory signal sequence of E2 [50]. Since the eukaryotic secretory signal sequence for E2 is actually part of the E1 sequence and not included in the E2 sequence, eukaryotic secretory signal sequences, such as the IgG κ signal sequence, have been used to transport E2 across the ER to maintain E2 in a glycosylated form as the native E2 protein [9, 15, 42, 51, 52]. In our study, the IgG κ signal sequence was fused to the N-terminus of GBV-C E2 to form the IgG E2 expression construct. E2DMID, an internal membrane interaction domain (MID) deletion mutant of IgG-E2, which lacks the ability to inhibit Gag processing [17], was used as a negative control in this study. All of the E2 constructs were Myc-tagged. The Gag-Pol expression vector (pGPCINS) was constructed using the VR1012 vector, a CMV promoter-based expression vector. In addition, the Gag-Pol gene was codon-optimized for Rev-independent expression in mammalian cells. The expression constructs ts045-VSVG-GFP (Addgene 11912) [53], pcDNA3-ARF1-HA (Addgene 10830) [54] and ARF1-GFP (Addgene 39554) [55] were obtained through Addgene. Note that although pcDNA3-ARF1-HA expresses bovine ARF1, it has an identical amino acid sequence to human ARF1.

Anti-p24 monoclonal antibody (Mab) (183-H12-5C) [56] and polyclonal anti-HIV-1 p17 (VU47) antibody [57] were obtained from the NIH AIDS Research and Reference Reagent Program (NIH-ARRRP). Other antibodies were obtained commercially, including anti-cMyc Mab 4A6 (Millipore), anti-HA Mab HA11 (Covance), anti-His Mab (GenScript), anti-calnexin C-20 antibody (Santa Cruz Biotechnology) and anti-TGN38 H-300 (Santa Cruz Biotechnology) antibody. Secondary antibodies included Alexa 488-conjugated goat anti-rabbit (Life Technologies), Alexa 546-conjugated donkey anti-mouse (Life Technologies), Alexa 546-conjugated donkey anti-goat (Life Technologies), Alexa 546-conjugated goat anti-rabbit (Life Technologies), Dyligth 488-conjugated donkey anti-mouse (Jackson ImmunoResearch Lab) and Dyligth 649-conjugated donkey anti-mouse (Jackson ImmunoResearch Lab) immunoglobulins. MG132, calpain inhibitor I (LLnL), Brefeldin A (BFA) and cycloheximide (CHX) were purchased from Sigma. Bortezomib was purchased from Selleck Chemicals. VECTASHIELD Antifade Mounting Medium with DAPI (H1200) was purchased from Vector Laboratories.

### Cell culture, virus-like particle (VLP) preparation and western blotting analysis

Human embryonic kidney (HEK) 293T and HeLa cells were cultured in DMEM containing 10% fetal bovine serum (FBS) at 37°C with 5% CO_2_ atmosphere. Plasmid DNA transfection was carried out with polyethylenimine (PEI) as described previously [58], which usually achieves more than 80% transfection rate in 293T cells (Figure S2). VLP preparation and Western blotting analysis were performed as previously described [49, 59]. Thirty micrograms of total protein from each cell lysate sample were typically loaded per lane of an SDS-PAGE gel for Western blotting analysis. Bio-Rad Precision Plus Protein Standard (Bio-Rad, 161-0375) was used as a protein molecular weight (MW) marker. The MW values are labeled on some of the Western blot images as 50, 37 and 25, which represent 50 KD, 37 KD and 25 KD, respectively.

### Real-time PCR

The ARF1-HA expression vector was co-transfected into 293T cells with either VR1012, E2, IgG-E2 or E2DMID expression vectors. Forty-eight hours post-transfection, cells were washed with cold phosphate-buffered saline (PBS), and total cellular RNA was isolated with the Aurum total RNA mini kit (Bio-Rad). The same amount of RNA was used to synthesize cDNA using the Reverse Transcriptase Core Kit (Eurogentec) following the method recommended by the manufacturer. Real-time PCR assays were performed using a CFX96 Real-time System (Bio-Rad) with iQ SYBR Green Supermix (Bio-Rad). The primer sequences for endogenous human *ARF1* were (forward) 5′-GTGACCACCATTCCCACCATAG-3′ and (reverse) 5′-TCATTGCTGTCCACCACGAAG-3′ as described elsewhere [60]. The primer sequences for the exogenous bovine *ARF1* were (forward) 5′-GGGAAAGACCACCATCCTGTA-3′ and (reverse) 5′-CACGTTGAAGCCATAGTGGGAAT-3′, and those for the internal control *glucuronidase, beta* (*GUSB*) gene were (forward) 5′-AAACGATTGCAGGGTTTCAC-3′ and (reverse) 5′-CTCTCGTCGGTGACTGTTCA-3′. The real-time PCR conditions were 95°C for 5 min, 40 cycles of 94°C for 15 sec and 60°C for 1 min, followed by melting curves generated from 65°C to 95°C, at increments of 0.5°C for 0.05 sec. The real-time PCR results were normalized using *GUSB* amplification levels and calculated by the 2^−ΔΔCT^ comparative method. All experiments were performed at least three separate times.

### CHX chase assay

Protein synthesis inhibitor CHX was used to study the half-life of ARF1-HA in the presence of the VR1012 or expression vectors for E2, IgG-E2 or E2DMID as previously described [61]. Twenty-four hours after the ARF1-HA or the E2 expression constructs were transfected into 293T cells, the cells were treated with CHX (200 μg/ml) to inhibit protein translation. The cells were harvested at the indicated time points and subsequently analyzed by Western blotting.

### Immunofluorescence microscopy

Confocal analysis was performed as described previously [62, 63]. In brief, HeLa cells were plated on glass coverslips in a 6-well plate and grown overnight. Cells were then transfected with indicated plasmids using Lipofectamine 2000. For analysis, cells were fixed with 4% paraformaldehyde in PBS solution at room temperature for 10 min, permeabilized with 0.1% Triton X-100 for 10 min and blocked with 5% bovine serum albumin overnight at 4°C. Immunofluorescent staining was performed using polyclonal anti-HIV-1 p17 VU47 (1:1,000), anti-cMyc Mab 4A6 (1:1,000), anti-calnexin antibody (1:50) and anti-TGN-38 antibody (1:50) as primary antibodies, which were detected by 488-conjugated goat anti-rabbit, 488-conjugated donkey anti-mouse, 546-conjugated donkey anti-goat, 546 goat anti-rabbit or 649 conjugated donkey anti-mouse secondary antibodies. Images were captured using a Nikon A1R confocal microscope.

### Ts045-VSVG-GFP transport assay

The assay was performed by following the method described by Volpicelli-Daley et al. [34]. Briefly, HeLa cells were plated on glass coverslips and cultured overnight. The expression vector ts045-VSVG-GFP was co-transfected with VR1012, E2, IgG-E2 or E2DMID into the HeLa Cells using Lipofectamine 2000. Six hours post-transfection, the medium was replaced with fresh medium. After incubation at 40°C for 16 h, cells were switched to 32°C for 90 min and then fixed. Immunofluorescent staining and confocal microscopy analysis were performed as described above.

### shRNA-mediated RNA interference of human proteasome subunit alpha type 1 (PMSA1)

The following oligos 5′-GATCCCCGACCACTGCCTGTGTCTCGTCTTGTATCTTCAAGAGAGATACAAGACGAGACACAGGCAGTGGTCTTTTTA-3′ and 5′-AGCTTAAAAAGACCACTGCCTGTGTCTCGTCTTGTATCTCTCTTGAAGATACAAGACGAGACACAGGCAGTGGTCGGG-3′ were annealed in annealing buffer (100 mM NaCl and 50 mM HEPES pH 7.4) and then cloned into the pRS-RO vector (OriGene Technologies) to generate the shRNA-PSMA construct. shRNA-PSMA or pRS-RO (as a negative control) was co-transfected with ARF1-HA and with either VR1012 or IgG-E2 into 293T cells. At 48 hours post-transfection, cells were harvested and subsequently analyzed by Western blotting.

### Membrane-binding analysis

Membrane flotation analysis of cell lysates was performed as detailed by Ono et al. [64]. In brief, 2 days after transfection, 293T cells were washed with PBS and disrupted with homogenization buffer containing 10% sucrose, 10 mM Tris-HCl (pH 7.5) and 1 mM Tris-ethylenediaminetetraacetic acid (TE) with Complete Protease Inhibitor Cocktail (Roche). Lysis was performed by sonication (twice for 15s each time). Nuclear fractions and unlysed cells were removed by adjusting the samples to 150 mM NaCl and 1 mM MgCl_2_ prior to centrifugation at 1,000 × *g* for 10 min at 4°C. Subsequently, 200 μl of the postnuclear supernatant was mixed with 800 μl of 85.5% sucrose in TE to adjust the sample to 73% sucrose. The sample was then placed at the bottom of a centrifuge tube and layered with 3 ml of 65% sucrose in TE and 1 ml of 10% sucrose in TE. The sucrose step gradient was centrifuged at 35,000 rpm for 18 h at 4°C using a Beckman SW55i rotor.

### Cytotoxicity assay

293T cells were transfected with 1 μg VR1012 or expression vector E2, IgG-E2 or E2DMID in a 12-well tissue culture plate. At 3 days and 7 days post-transfection, cells were harvested for Western blotting analysis and stained using the LIVE/DEAD^®^ Cell Vitality Assay kit (Life Technologies, L34951) to measure cytotoxicity following the product manual. The stained cells were analyzed using a BD Biosciences FACScalibur with excitation at 488 nm, and the fluorescence emission was measured at 530 nm and 575 nm.

### Statistical analysis

All experiments were independently repeated at least three times, and representative data are shown. Error bars indicate standard deviation. Statistical analysis was performed using GraphPad Prism 5 (GraphPad Software). Comparisons between two groups were carried out using two-sided Student's *t*-tests. Comparisons between more than two groups were conducted using one-way analysis of variance (ANOVA). Significance was defined as a *P*-value < 0.05. Statistical tests used for each data group are described in the figure legends.

## SUPPLEMENTARY MATERIAL FIGURES


