# Supplementary material for: GB virus type C E2 protein inhibits human immunodeficiency virus type 1 Gag assembly by downregulating human ADP-ribosylation factor 1
Source: Oncotarget. 2015 Dec 9;6(41):43293–309. doi: 10.18632/oncotarget.6537 (PMC4791233; doi:10.18632/oncotarget.6537)
Supplement: Supplementary file 1 [file oncotarget-06-43293-s001.pdf]

GB virus type C E2 protein inhibits human immunodeficiency virus type 1 Gag assembly by downregulating human ADP-ribosylation factor 1

Supplementary Material

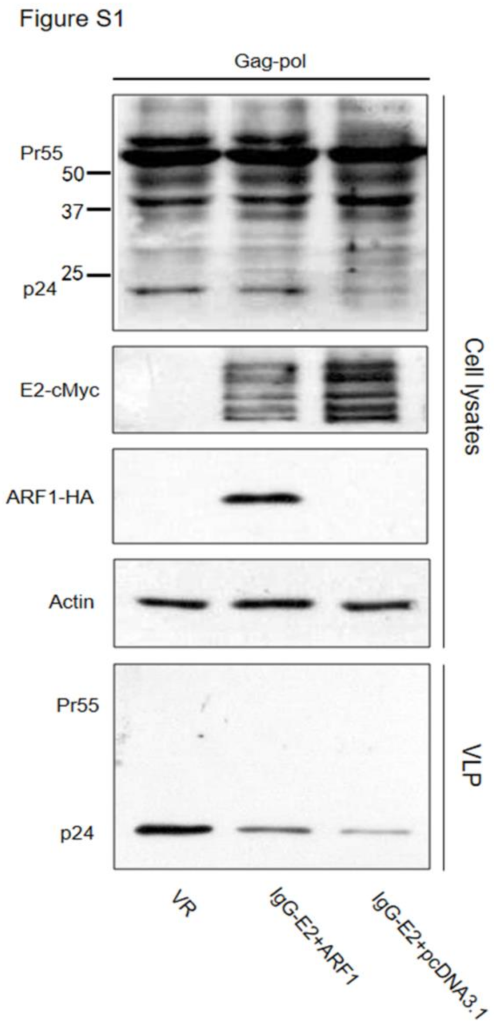

Figure S1. Maintaining ARF-1 expression compromises the inhibitory effect of GBV-C E2 on HIV-1 Gag processing in Jurkat T cells. The Gag-Pol expression vector was co-transfected with VR1012, IgG-E2 or IgG-E2 plus ARF1-HA expression construct into Jurkat T cells by using Lonza amaxa nucleofector II system. Cells were subjected to Western blotting analysis, and the culture supernatant was subjected to ultracentrifugation for measuring VLP release.

Figure S2

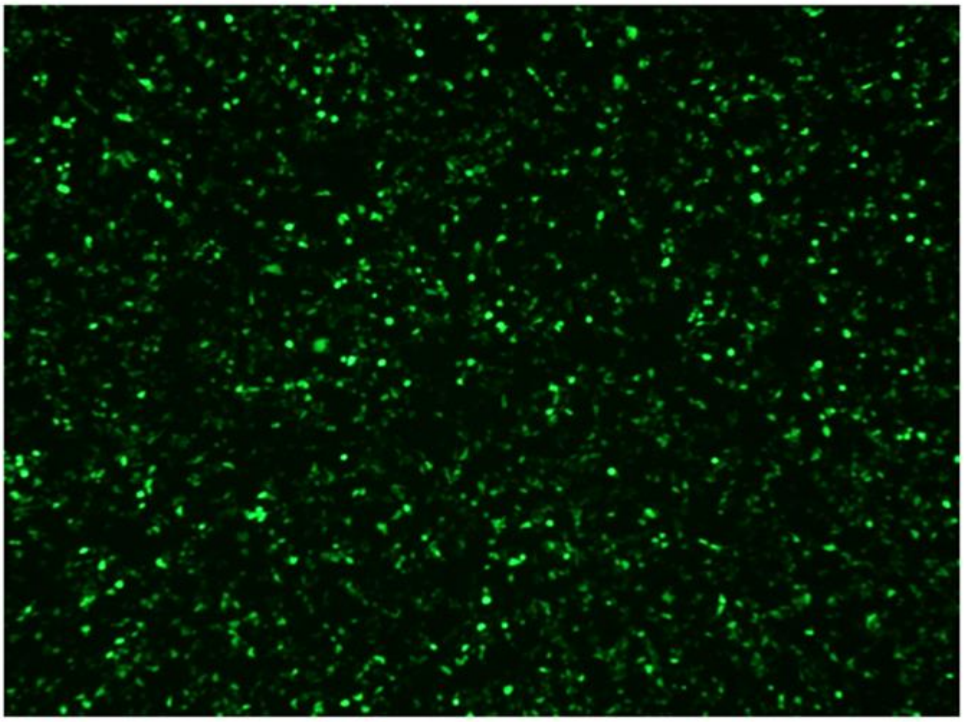

pcDNA3.1-GFP

Figure S2. The efficient transfection of plasmid DNA by PEI method. pcDNA-eGFP construct was transfected into 293T cells using PEI transfection method mentioned in the material and methods section. Forty-eight hours post-transfection, the picture was taken using fluorescent microscope.

Figure S3

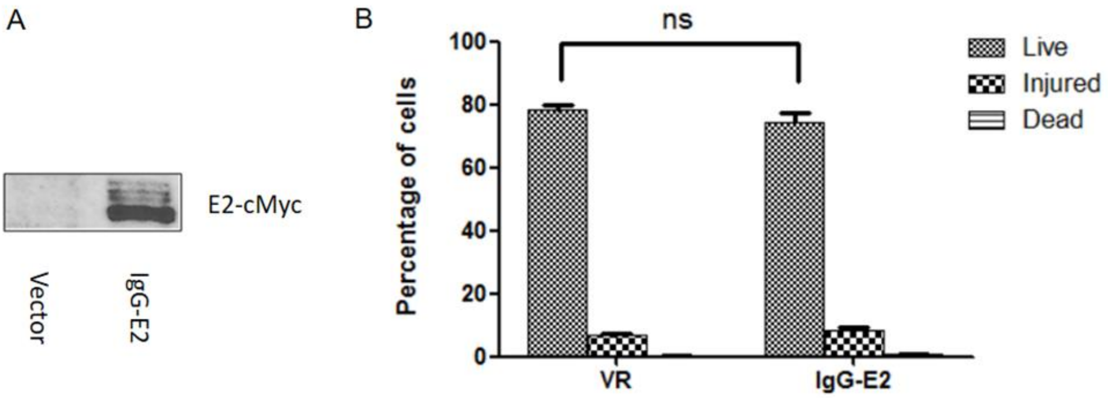

Figure S3. Expression of IgG-E2 does not cause dramatic cytotoxicity in SupT1 T cells.

A. SupT1 cells were transfected with VR1012, IgG-E2 using Neon transfection system (Invitrogen). At 3 days post-transfection, cells were harvested for Western blotting analysis. **B**. Transfected cells were also stained by using the LIVE/DEAD® Cell Vitality Assay kit. Cell viability was analyzed using a BD Biosciences FACScalibur with excitation at 488 nm, and the fluorescence emission was measured at 530 nm and 575 nm. The data are represented as mean ± SD, ns indicates no significant difference, Student's t-test. All the values are from the average of four independent experiments.
